# Epidemiological characteristics of severe fever with thrombocytopenia syndrome and its relationship with meteorological factors in Liaoning Province, China

**DOI:** 10.1186/s13071-022-05395-4

**Published:** 2022-08-06

**Authors:** Zijiang Wang, Shiting Yang, Li Luo, Xiaohao Guo, Bin Deng, Zeyu Zhao, Jia Rui, Shanshan Yu, Bin Zhao, Yifang Wang, Jingyi Chen, Yingwei Sun, Tianmu Chen, Xinyu Feng

**Affiliations:** 1 Liaoning Provincial Center for Disease Control and Prevention, Shayang Road No. 242, Heping District, Shenyang, Liaoning People’s Republic of China; 2 State Key Laboratory of Molecular Vaccinology and Molecular Diagnostics, School of Public Health, Xiamen University, 4221-117 South Xiang’an Road, Xiang’an District, Xiamen, Fujian People’s Republic of China; 3 Laboratory Department, State Key Laboratory of Molecular Vaccinology and Molecular Diagnostics, Xiang’an Hospital of Xiamen University, Xiamen, Fujian People’s Republic of China; 4 School of Informatics, Xiamen University, Xiang’an Road, Xiang’an District, Xiamen, Fujian People’s Republic of China; 5 School of Medicine, Nankai University, Weijin Road No. 94, Nankai District, Tianjin, People’s Republic of China; 6 Chinese Center for Disease Control and Prevention (Chinese Center for Tropical Diseases Research), NHC Key Laboratory of Parasite and Vector Biology, National Institute of Parasitic Diseases, WHO Collaborating Centre for Tropical Diseases, National Center for International Research On Tropical Diseases, Joint Research Laboratory of Genetics and Ecology On Parasite-Host Interaction, Chinese Center for Disease Control and Prevention & Fudan University, NO. 207 Ruijin 2nd Road, Huangpu District, Shanghai, People’s Republic of China; 7 School of Global Health, Chinese Center for Tropical Diseases Research, Shanghai Jiao Tong University School of Medicine, One Health Center, Shanghai Jiao Tong University-The University of Edinburgh, Shanghai, People’s Republic of China; 8 Department of Biology, College of Life Sciences, Inner Mongolia University, Hohhot, People’s Republic of China

**Keywords:** Epidemiological characteristics, Severe fever with thrombocytopenia syndrome, Meteorological factors

## Abstract

**Background:**

Severe fever with thrombocytopenia syndrome (SFTS), one kind of tick-borne acute infectious disease, is caused by a novel bunyavirus. The relationship between meteorological factors and infectious diseases is a hot topic of current research. Liaoning Province has reported a high incidence of SFTS in recent years. However, the epidemiological characteristics of SFTS and its relationship with meteorological factors in the province remain largely unexplored.

**Methods:**

Data on reported SFTS cases were collected from 2011 to 2019. Epidemiological characteristics of SFTS were analyzed. Spearman’s correlation test and generalized linear models (GLM) were used to identify the relationship between meteorological factors and the number of SFTS cases.

**Results:**

From 2011 to 2019, the incidence showed an overall upward trend in Liaoning Province, with the highest incidence in 2019 (0.35/100,000). The incidence was slightly higher in males (55.9%, 438/783), and there were more SFTS patients in the 60–69 age group (31.29%, 245/783). Dalian City and Dandong City had the largest number of cases of SFTS (87.99%, 689/783). The median duration from the date of illness onset to the date of diagnosis was 8 days [interquartile range (IQR): 4–13 days]. Spearman correlation analysis and GLM showed that the number of SFTS cases was positively correlated with monthly average rainfall (*r*_*s*_ = 0.750, *P* < 0.001; *β* = 0.285, *P* < 0.001), monthly average relative humidity (*r*_*s*_ = 0.683, *P* < 0.001; *β* = 0.096, *P* < 0.001), monthly average temperature (*r*_*s*_ = 0.822, *P* < 0.001; *β* = 0.154, *P* < 0.001), and monthly average ground temperature (*r*_*s*_ = 0.810, *P* < 0.001; *β* = 0.134, *P* < 0.001), while negatively correlated with monthly average air pressure (*r*_*s*_ = −0.728, *P* < 0.001; *β* = −0.145, *P* < 0.001), and monthly average wind speed (*r*_*s*_ = −0.272, *P* < 0.05; *β* = −1.048, *P* < 0.001). By comparing both correlation coefficients and regression coefficients between the number of SFTS cases (dependent variable) and meteorological factors (independent variables), no significant differences were observed when considering immediate cases and cases with lags of 1 to 5 weeks for dependent variables. Based on the forward and backward stepwise GLM regression, the monthly average air pressure, monthly average temperature, monthly average wind speed, and time sequence were selected as relevant influences on the number of SFTS cases.

**Conclusion:**

The annual incidence of SFTS increased year on year in Liaoning Province. Incidence of SFTS was affected by several meteorological factors, including monthly average air pressure, monthly average temperature, and monthly average wind speed.

**Graphical Abstract:**

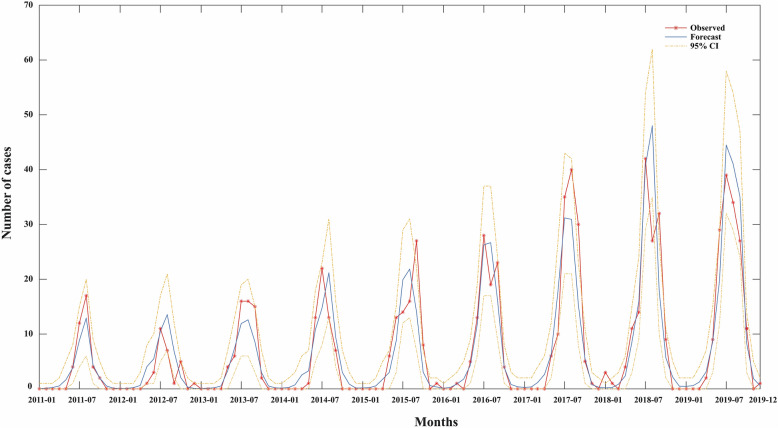

**Supplementary Information:**

The online version contains supplementary material available at 10.1186/s13071-022-05395-4.

## Background

Severe fever with thrombocytopenia syndrome (SFTS) is an acute infectious disease characterized by human-to-human transmission and a high fatality rate [1]. The SFTS virus (SFTSV), the causative agent of the disease, is of the *Bunyaviridae* family and contains a negative-sense RNA genome consisting of three fragments [2, 3]. SFTSV has been detected in several tick species, including *Haemaphysalis longicornis*, *Rhipicephalus microplus*, *Amblyomma testudinarium*, and *Ixodes nipponensis*, of which *H. longicornis* is the most important vector for SFTSV transmission. *Haemaphysalis longicornis* is widely distributed in the Pacific coastal region, including areas of high SFTS incidence in China [4, 5]. People may become infected with SFTSV after being bitten by ticks [6, 7], and SFTSV can be transmitted from person to person through direct contact with body fluids or blood [8, 9]. The most common clinical symptoms are fever, thrombocytopenia, headache, nausea, diarrhea, and gingival and conjunctival hemorrhage. The disease may progress rapidly in some patients, leading to severe complications, such as multiple organ failure, and even death [10].

SFTS is classified as a class B notifiable infectious disease in China. The first human cases of SFTS were reported in northeast and central China in 2009 [11], followed by South Korea, Japan, and the United Arab Emirates [12, 13]. Moreover, a genetically related virus of SFTSV has been isolated in the United States [14]. The areas of high SFTS incidence in China are mainly concentrated in Henan, Hubei, Shandong, Anhui, Liaoning, Jiangsu, and Zhejiang provinces [15], especially in rural areas in mountainous and hilly regions, presenting a highly spatial feature. So far, approximately 90% of the cases reported worldwide are from China [2], and SFTS has recently become a severe public health problem in China. Therefore, it is important to clarify the epidemiological characteristics of SFTS for its prevention and control both in China and worldwide.

SFTSV is usually transmitted to humans through tick bites. Studies have constructed disease transmission models and found that cutting off the environmental and animal–host transmission routes reduces the cumulative number of SFTS cases, confirming that the environment is also an important transmission route [16]. It has been shown that meteorological factors can influence the survival and reproduction of SFTSV and the growth dynamics of tick vectors, and can directly affect the outdoor activities of humans [17]. So far, studies have investigated the influence of meteorological factors on SFTSV infection by constructing logistic geographically weighted models, distributed lag nonlinear models (DLNMs), and ecological niche modeling of maximum entropy (MaxEnt). The results show that the incidence of SFTS is related to temperature, precipitation, relative humidity, sunshine duration, and air pressure [18–21]. Still, no previous study has examined the influence of ground temperature on SFTS. Due to the specificity of the climate and population density in Liaoning Province, we analyzed the epidemiological characteristics of SFTS in the province and constructed a generalized linear model (GLM) to systematically and comprehensively explore the correlation between meteorological factors and SFTS incidence. This research aimed to provide epidemiological evidence for areas in which tick surveillance is not conducted.

## Methods

### Ethics statement

This disease control effort was part of the Center for Disease Control and Prevention (CDC)’s routine responsibility in Liaoning Province, China, and as such, institutional review board approval and informed consent were not required. All data analyzed were anonymized.

### Study area

Liaoning Province (38°43′N–43°26′ N, 118°53′ E–125°46′ E) is located in the southern part of northeastern China. It has a population of 43.517 million and an area of 148,600 km^2^. Liaoning Province has 14 prefecture-level cities, including Shenyang, Dalian, Anshan, Fushun, Benxi, Dandong, Jinzhou, Yingkou, Fuxin, Liaoyang, Panjin, Tieling, Chaoyang, and Huludao. The province is characterized by a temperate monsoon climate, with an annual average temperature of 7–11 °C and annual precipitation of 600–1100 mm.

### Date collection

The SFTS case data for the study area from January 2011 to December 2019 were obtained from the Liaoning Provincial Disease Prevention and Control Information System. The SFTS case information included the name, sex, age, address, occupation, onset date, and diagnosis date. The meteorological data from January 2011 to December 2019 were derived from the Meteorological Data Center of the Chinese Meteorological Administration and included data on temperature, relative humidity, air pressure, rainfall, sunshine duration, wind speed, and ground temperature. Monthly meteorological data were obtained from the average of 27 meteorological stations at the prefecture or city level in Liaoning Province. The daily number of cases (unlagged ones) was defined as “immediate cases.”

### Statistical analysis

The collected data were coded and entered in Microsoft Excel 2013 (Microsoft Corp., USA), and statistical analysis was performed using SPSS 13.0 software (IBM Corp., Armonk, NY, USA) and customized MATLAB software (The MathWorks, Natick, MA, USA). The correlation between various meteorological factors and the number of SFTS cases was determined using the Spearman correlation test. The relationship between SFTS cases and meteorological factors was assessed using GLMs.

As the Poisson distribution is frequently used to describe phenomena that have few positive outcomes over many trials [22], we assume that the conditional distribution of the monthly number of cases when the meteorological factor is given follows a Poisson distribution:

$$\{Y|X=x\}\sim Poisson\left({\mathbb{E}}\{Y|X=x\}\right)$$

where *Y* denotes the monthly number of SFTS cases, and *X* denotes one of seven meteorological factors.

Assuming the expectation of $\{Y|X=x\}$ satisfies

$$ln\left({\mathbb{E}}\{Y|X=x\}\right)=a+bx$$

where $ln\left(\right)$ denotes the natural logarithm, $a$ and $b$ are parameters to be fitted.

The parameter fitting is carried out using the maximum likelihood estimation (MLE) described.

The relationship between the number of SFTS cases and meteorological factors was investigated using a GLM. The meteorological factors associated with the number of SFTS cases were selected based on stepwise regression [starting with an initial constant model, and searching for terms to add and to remove based on the sum of squared errors (SSE) in each step, see MATLAB document of the function stepwiseglm for more details], and these meaningful variables were incorporated into the construction of the model. Data from 2011 to 2019 in Liaoning Province were randomly divided into five sets of SFTS and meteorological data, with one fifth of the data serving as the training set and the remaining four fifths of the data serving as the test set. Random cross-validation was performed 100 times to test the accuracy and robustness of the model through the distribution of intraclass correlation coefficient (ICC) with the *F*-test [23, 24]. A *P*-value < 0.05 indicates that the difference in the tests is statistically significant.

## Results

### Epidemiological characteristics of SFTS

**Fig. 1:**
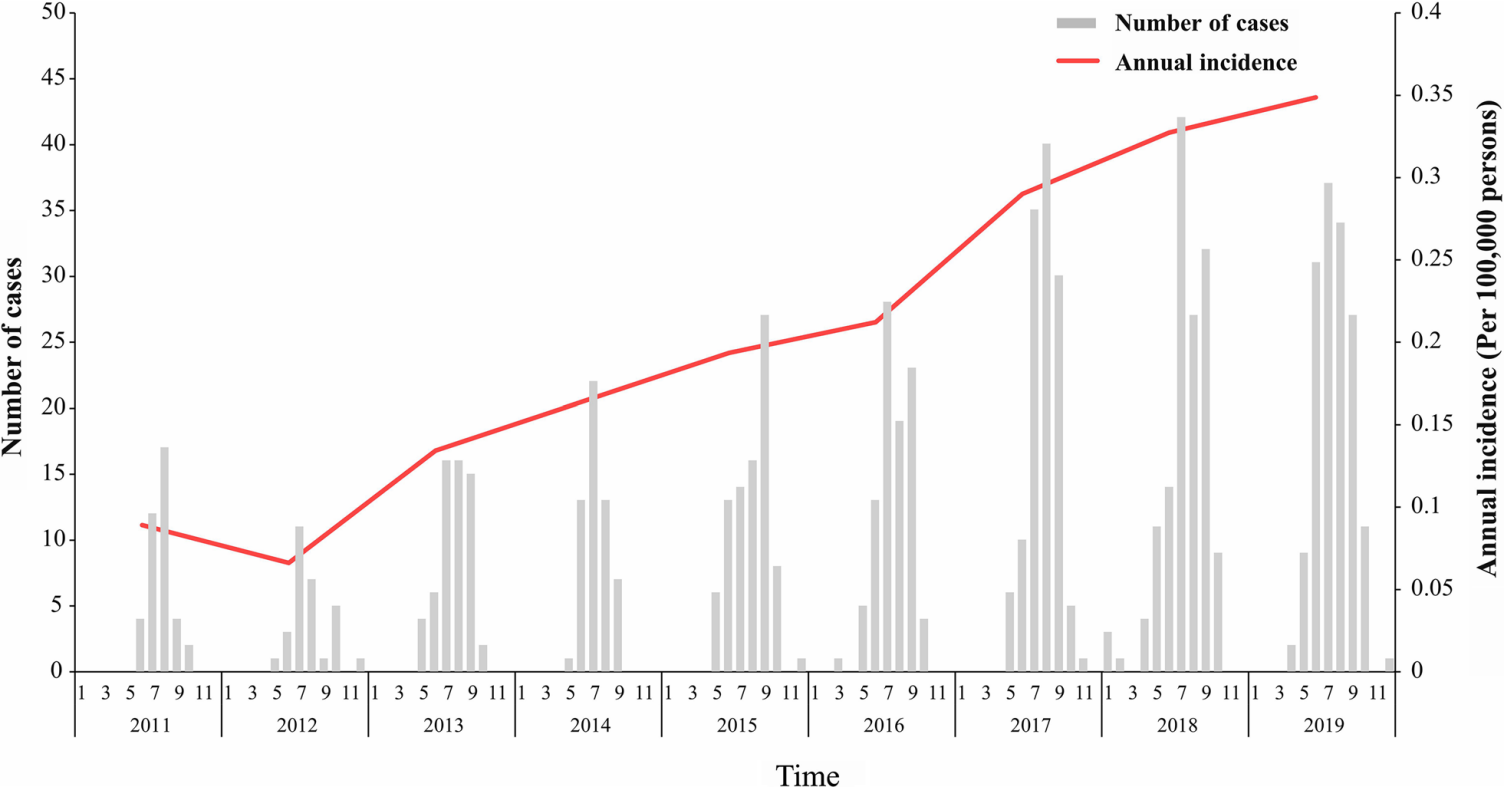
Annual incidence trends and monthly distribution in Liaoning Province, China, 2011–2019

We collected 783 cases of SFTS reported in Liaoning Province from 2011 to 2019, including 23 deaths, with an average annual incidence of 0.20/100,000 and an average case fatality rate of 2.94%. From 2011 to 2019, the incidence showed an overall upward trend, with the highest incidence in 2019 (0.35/100,000). The incidence of SFTS showed prominent seasonal characteristics. The reported cases of SFTS mainly occurred between May and October, accounting for 98.08% of all cases, and peaked in July and August (Fig. 1).

**Fig. 2:**
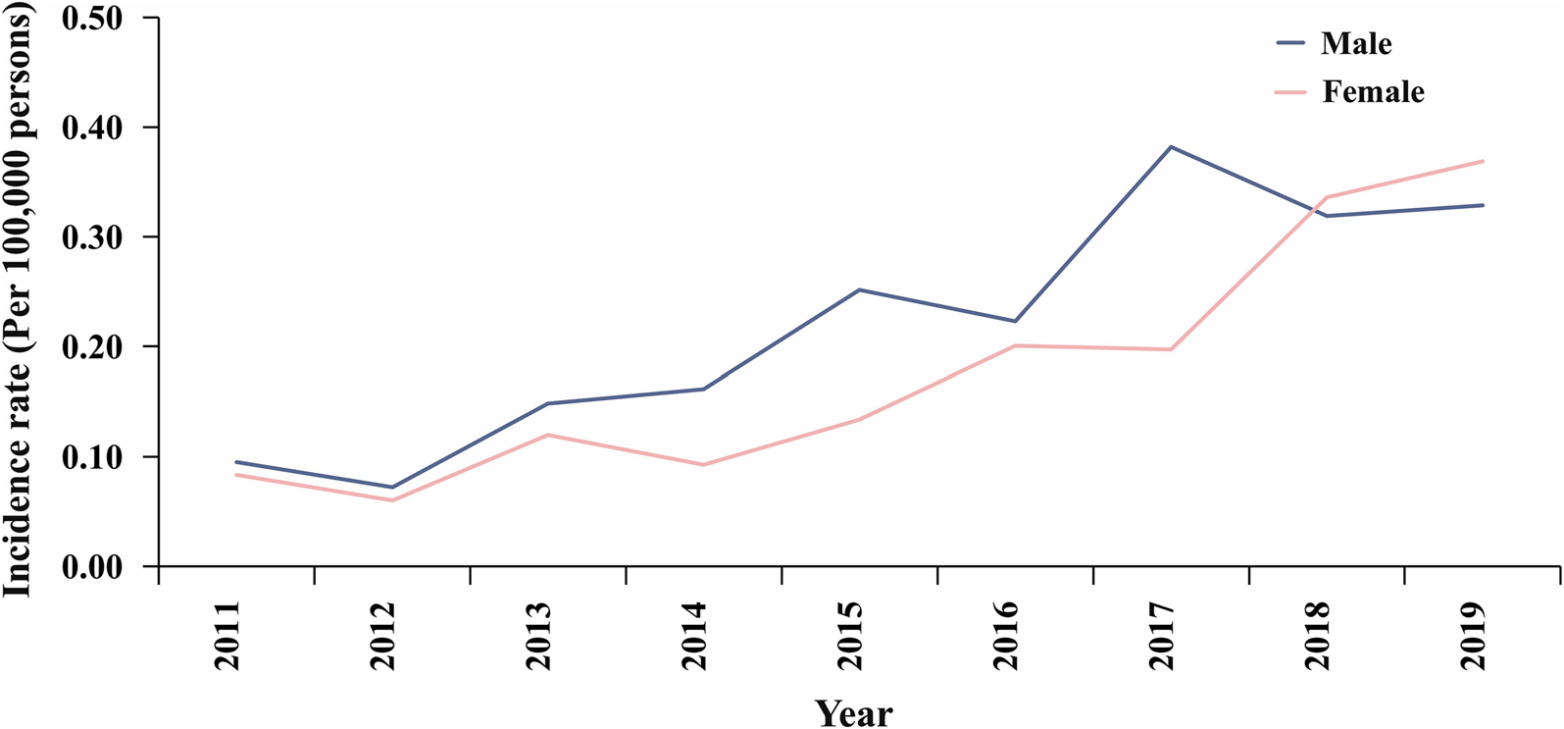
Gender distribution of SFTS cases, Liaoning Province, China, 2011–2019

Among the 783 reported SFTS cases, 438 were in males (55.9%, 438/783) and 345 cases were in females (44.1%, 345/783). Between 2011 and 2019, the overall increasing trend in incidence was observed for both males and females (Fig. 2). The age distribution of patients with SFTS from 2011 to 2019 ranged from 1 to 89 years old, with an average age of 60.35 years. The cases were concentrated in the 60–69 age group, which accounted for 31.29% (245/783) of all included cases (Table 1). Most cases of SFTS were in farmers (65.52%, 513/783), followed by homemakers and unemployed people (25.42%, 199/783) (Table 1).

**Table 1:** Epidemiological characteristics of 783 reported SFTS human cases, Liaoning Province, China, 2011–2019

Variables	Number of cases	Percentage (%)
Sex	783	100.00
Male	438	55.94
Female	345	44.06
Age (years)	783	100.00
0–9	2	0.26
10–19	4	0.51
20–29	9	1.15
30– 39	28	3.58
40–49	96	12.26
50–59	208	26.56
60–69	245	31.29
70–79	158	20.18
> 80	33	4.21
Occupation	783	100.00
Farmer	513	65.52
Housework and unemployment	199	25.42
Retired	21	2.68
Unknown	22	2.81
Worker	4	0.51
Cadre staff	7	0.89
Catering industry	1	0.13
Teacher	2	0.26
Migrant worker	2	0.26
Herdsman	2	0.26
Student	4	0.51
Diaspora children	2	0.26
Medical staff	2	0.26
Other: self-employed	1	0.13
Others	1	0.13

**Fig. 3:**
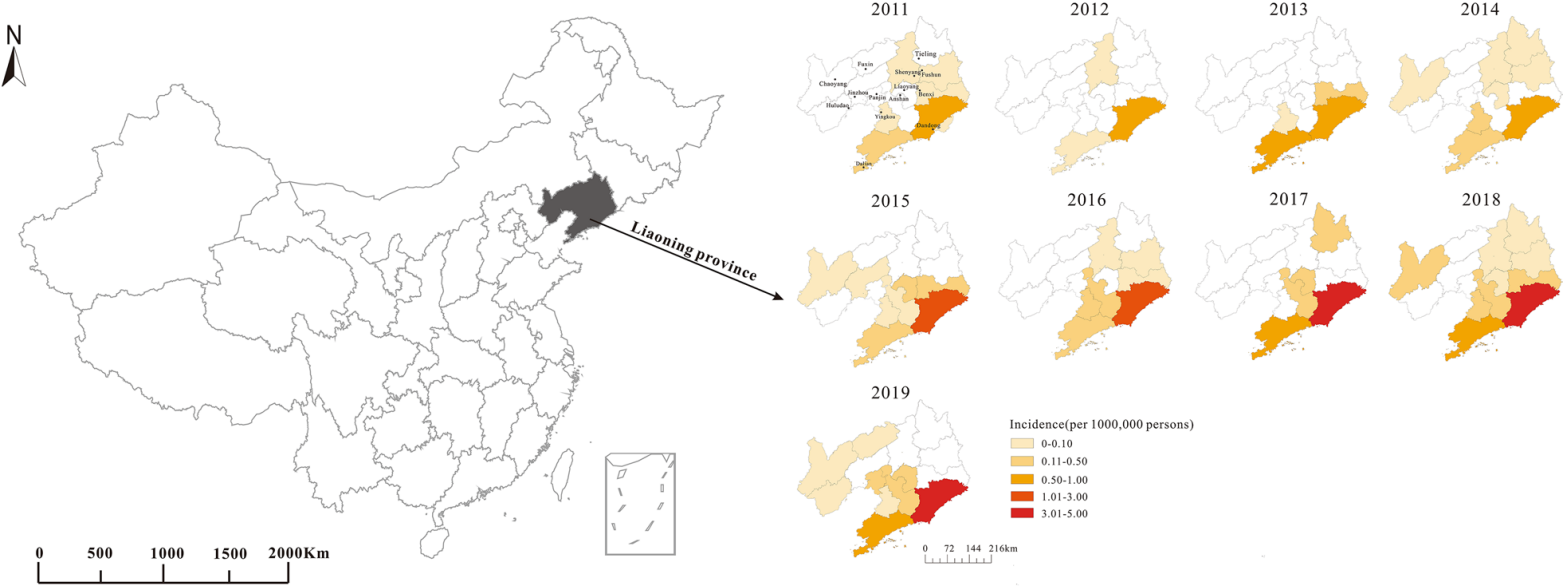
Geographical distribution of SFTS cases, Liaoning Province, China, 2011–2019

From 2011 to 2019, 87.99% of the SFTS cases were reported in Dalian City and Dandong City, accounting for 32.69% and 55.30%, respectively, while there were only 94 SFTS cases in the other 12 prefecture-level cities. The average annual incidence in Dandong was the highest (1.99/100,000) during the study period and showed an increasing trend. In contrast, Jinzhou City had the lowest average annual incidence (0.0036/100,000), and no SFTS cases were reported in Fuxin City, Panjin City, or Huludao City during 2011–2018 (Fig. 3).

**Fig. 4:**
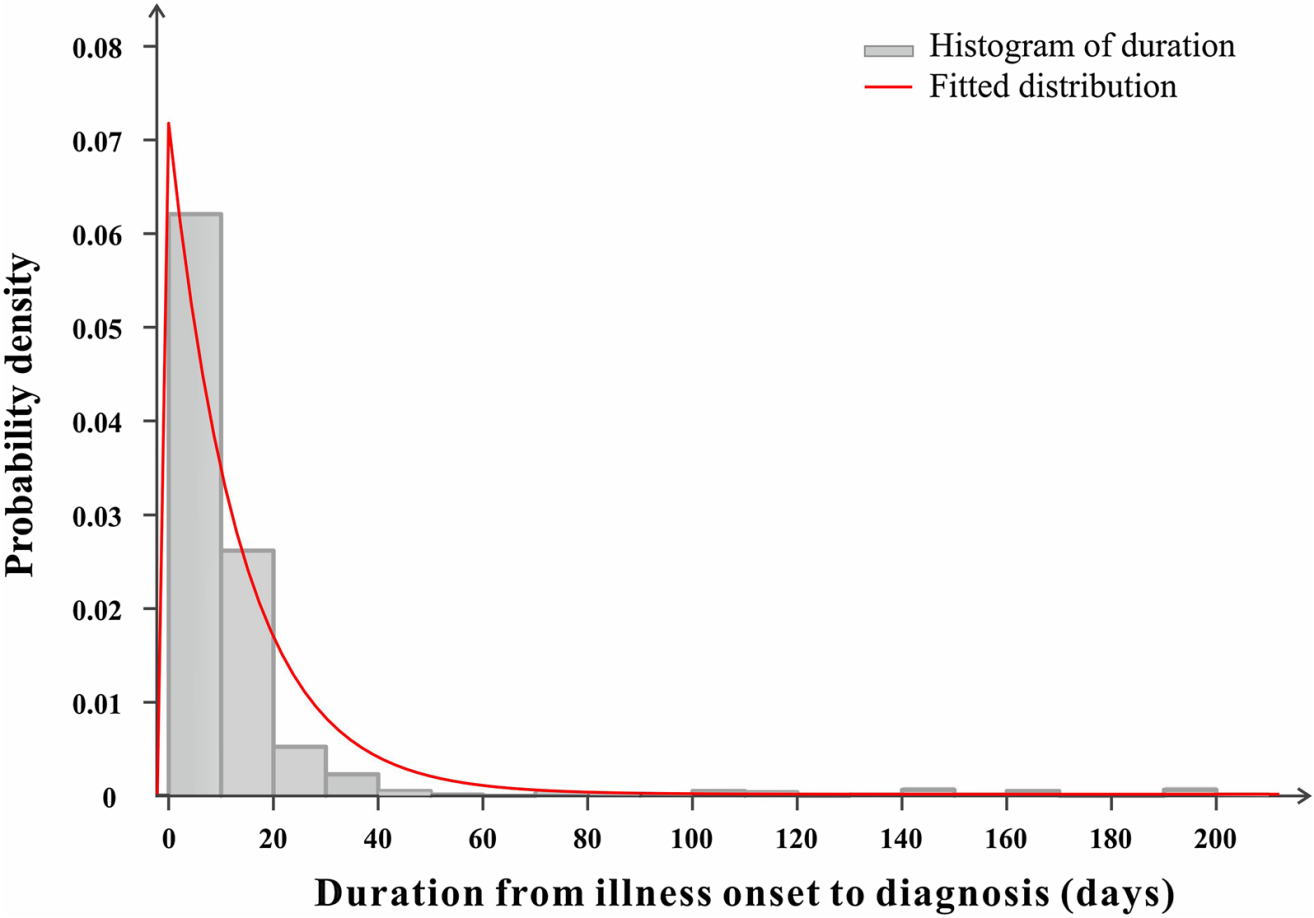
Distribution of duration from illness onset date to the diagnosed date of 783 SFTS cases, Liaoning Province, China, 2011–2019

The median duration of SFTS from the date of onset to the date of diagnosis was 8 days [interquartile range (IQR): 4–13 days]. The date of diagnosis mainly occurred within 0–10 days, accounting for 65.64% (Fig. 4), while those diagnosed within 0–5 days accounted for 32.69%.

### Correlation between meteorological factors and the number of SFTS cases

In Liaoning Province, the incidence of SFTS often reached a peak in July, with average annual meteorological factors as follows: air pressure 992.176 hPa, relative humidity 77.35%, sunshine duration 7.6 h/day, temperature 24.81 °C, wind speed 2.35 m/s, and ground temperature 28.89 °C. Spearman correlation analysis showed that the number of SFTS cases was positively correlated with monthly average rainfall (*r*_s_ = 0.750, *P* < 0.001), monthly average relative humidity (*r*_s_ = 0.683, *P* < 0.001), monthly average temperature (*r*_s_ = 0.822, *P* < 0.001) and monthly average ground temperature (*r*_s_ = 0.810, *P* < 0.001), while negatively correlated with monthly average air pressure (*r*_s_ = −0.728, *P* < 0.001), and monthly average wind speed (*r*_s_ = −0.272, *P* < 0.05) (Additional file 1: Figure S1). Considering the hysteresis effect of meteorological factors on diseases, we lagged the number of SFTS cases on a 1–5-week scale for further analysis. Spearman correlation analysis showed that the correlation between the lagged number of cases and meteorological factors showed no significant change compared to those in a real-time manner (Table 2).

**Table 2:** Comparison of Spearman's correlation coefficients between the number of immediate and lagged SFTS cases and meteorological factors

Meteorological factors	The number of SFTS cases											
	Immediate effect		One-week lag		Two-week lag		Three-week lag		Four-week lag		Five-week lag	
	*r*_*s*_	*P*	*r*_*s*_	*P*	*r*_*s*_	*P*	*r*_*s*_	*P*	*r*_*s*_	*P*	*r*_*s*_	*P*
Monthly average air pressure (hPa) (*X*_1_)	−0.728	*P* < 0.001	−0.681	*P* < 0.001	−0.611	*P* < 0.001	−0.538	*P* < 0.001	−0.727	*P* < 0.001	−0.683	*P* < 0.001
Monthly average relative humidity (%) (*X*_2_)	0.683	*P* < 0.001	0.707	*P* < 0.001	0.700	*P* < 0.001	0.687	*P* < 0.001	0.688	*P* < 0.001	0.708	*P* < 0.001
Monthly average sunshine duration (h) (*X*_3_)	−0.155	*P* > 0.001	0.063	*P* > 0.05	0.023	*P* > 0.05	−0.013	*P* > 0.05	0.145	*P* > 0.05	0.060	*P* > 0.05
Monthly average temperature (°C) (*X*_4_)	0.822	*P* < 0.001	0.790	*P* < 0.001	0.743	*P* < 0.001	0.694	*P* < 0.001	0.826	*P* < 0.001	0.795	*P* < 0.001
Monthly average wind speed (m/s) (*X*_5_)	−0.272	*P* < 0.05	−0.293	*P* < 0.05	−0.315	*P* < 0.05	−0.349	*P* < 0.001	−0.279	*P* < 0.05	−0.293	*P* < 0.05
Monthly average ground temperature(°C) (*X*_6_)	0.810	*P* < 0.001	0.773	*P* < 0.001	0.725	*P* < 0.001	0.673	*P* < 0.001	0.815	*P* < 0.001	0.777	*P* < 0.001
Monthly average rainfall (mm) (*X*_7_)	0.750	*P* < 0.001	0.750	*P* < 0.001	0.710	*P* < 0.001	0.674	*P* < 0.001	0.754	*P* < 0.001	0.748	*P* < 0.001

### Regression relationship between the number of SFTS cases and meteorological factors estimated using GLM

We constructed generalized linear regressions of the number of immediate cases and those lagged on a 1–5-week scale of case data (*Y*) on the following seven candidate factors: monthly average air pressure (*X*_1_), monthly average relative humidity (*X*_2_), monthly average sunshine duration (*X*_3_), monthly average temperature (*X*_4_), monthly average wind speed (*X*_5_), monthly average ground temperature (*X*_6_), and monthly average rainfall (*X*_7_). Based on the comparison of the *P*-values and the slope coefficients in both the lagged and un-lagged models, we concluded that these meteorological factors have no clear impact on SFTS incidence through time lag (Table 3).

**Table 3:** Comparison of slope coefficients in regressions of the number of immediate and lagged SFTS cases with meteorological factors

Meteorological factors	The number of SFTS cases											
	Immediate effect		One-week lag		Two-week lag		Three-week lag		Four-week lag		Five-week lag	
	*β*	*P*	*β*	*P*	*β*	*P*	*β*	*P*	*β*	*P*	*β*	*P*
Monthly average air pressure (hPa) (*X*_1_)	−0.145	*P* < 0.001	−0.126	*P* < 0.001	−0.101	*P* < 0.001	−0.086	*P* < 0.001	−0.144	*P* < 0.001	−0.124	*P* < 0.001
Monthly average relative humidity (%) (*X*_2_)	0.096	*P* < 0.001	0.096	*P* < 0.001	0.093	*P* < 0.001	0.089	*P* < 0.001	0.094	*P* < 0.001	0.095	*P* < 0.001
Monthly average sunshine duration (h) (*X*_3_)	0.026	*P* > 0.05	−0.022	*P* > 0.05	−0.033	*P* > 0.05	−0.058	*P* > 0.05	0.023	*P* > 0.05	−0.020	*P* > 0.05
Monthly average temperature (°C) (*X*_4_)	0.154	*P* < 0.001	0.138	*P* < 0.001	0.121	*P* < 0.001	0.102	*P* < 0.001	0.154	*P* < 0.001	0.138	*P* < 0.001
Monthly average wind speed (m/s) (*X*_5_)	−1.048	*P* < 0.001	−1.210	*P* < 0.001	−1.421	*P* < 0.001	−1.454	*P* < 0.001	−1.043	*P* < 0.001	−1.225	*P* < 0.001
Monthly average ground temperature (°C) (*X*_6_)	0.134	*P* < 0.001	0.119	*P* < 0.001	0.103	*P* < 0.001	0.086	*P* < 0.001	0.134	*P* < 0.001	0.119	*P* < 0.001
Monthly average rainfall (mm) (*X*_7_)	0.285	*P* < 0.001	0.281	*P* < 0.001	0.271	*P* < 0.001	0.257	*P* < 0.001	0.280	*P* < 0.001	0.274	*P* < 0.001

**Fig. 5:**
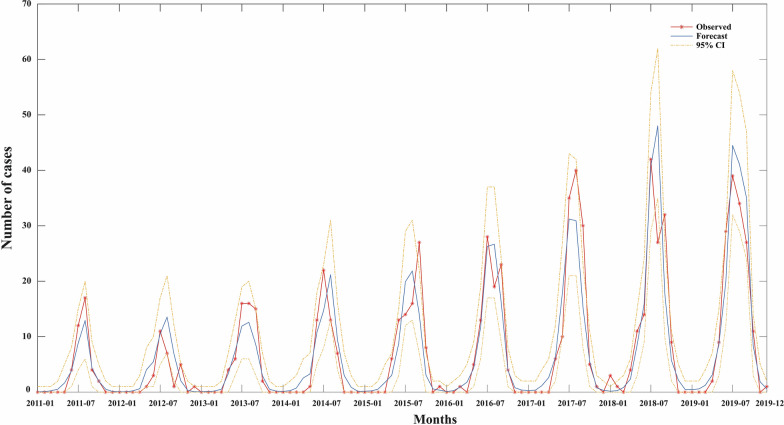
Fitting results of the optimal selected GLM. The *X*-axis represents the time sequence from January 2011 to December 2019; the *Y*-axis represents the monthly number of SFTS cases in Liaoning Province; the red line with stars represents the observed number of cases; the blue line represents model predictions according to the optimal model (including four factors: monthly average pressure, monthly average temperature, monthly average wind speed, time sequence), and the yellow lines represent the upper and lower bounds of the 95% confidence interval of predication

Considering the annually increasing trend of the incidence, we also included a variable of time sequence (*X*_8_, the cumulative month counts since the earliest month with all other meteorological factors) in the regression model. Stepwise regression was achieved by using a combination of both forward selection and backward elimination methods. We then constructed an optimal model that involves four selected variables (monthly average pressure, monthly average temperature, monthly average wind speed, and time sequence) (Fig. 5). The equation is shown as follows:

$$ln\left(E\left\{Y\mid {X}_{1}={x}_{1},{X}_{4}={x}_{4},{{X}_{5}={x}_{5},X}_{8}={x}_{8}\right\}\right)=-62.426+0.062{x}_{1}+ 0.182{x}_{4}-0.520{x}_{5}+0.017{x}_{8}$$

The model was cross-validated by 100 runs with a median ICC of 0.927 (Additional file 2: Figure S2) and an *F*-test value of 20.469 (*P* < 0.01).

## Discussion

SFTS seriously threatens human health and represents a significant economic burden to patients and their families [25]. Recently, the relationship between meteorological factors and infectious diseases has become a research hotspot in public health. Meteorological factors can affect disease occurrence, development, and transmission by affecting biological pathogens, vectors, and hosts [26–28]. In the current research, we aimed to clarify the epidemiological characteristics of SFTS in Liaoning Province and the meteorological factors associated with the occurrence of cases.

Although we found that the SFTS incidence was correlated with meteorological factors in Liaoning Province, our research still has some limitations. First, the case data in this study come from a passive surveillance system, and there may be some subclinical cases that have not been included. Second, apart from meteorological variables, social and economic factors may also affect the incidence of SFTS. Third, due to the lack of vector population, density, and distribution data, the correlation between intermediate hosts (ticks) and meteorological factors has not been explored in detail. The above limitations suggest that the relevant authorities should strengthen the screening of cases with subclinical symptoms of SFTS and the monitoring of vector data.

The incidence of SFTS in Liaoning Province showed an overall upward trend from 2011 to 2019, which may be explained by the enhanced public awareness of SFTS and the improvements in diagnostic clinical settings in China. As the life-cycle of a tick is sensitive to climate, warmer seasons are the active period for *H. longicornis* ticks, particularly July [29]. Our analysis indicated that the incidence of SFTS mainly occurred between May and October and reached its peak in July, with the peak incidence period coinciding with the active period of the tick.

SFTS was highly prevalent in people aged 60–69 years, and more than half of the patients were farmers, which is consistent with the study results in Anhui Province [30]. This finding may be explained by the higher likelihood of elderly individuals being exposed to ticks. Recently, the elderly have become the primary labor force in agriculture with high-speed urbanization in China. In contrast, the young generation move to large cities to look for job opportunities and leave the elders to engage in agricultural work such as plowing fields, mowing grass, and grazing [31]. Additionally, we noted that the majority of SFTS cases were male, which was also reflected in the research results for Xinyang, Henan [32]. Current evidence indicates potential links between the exposure risk and SFTS incidence and suggests interventions for high-risk populations.

Our results showed that from 2011 to 2019, 87.99% of SFTS cases in Liaoning Province were concentrated in two prefecture-level cities, Dalian and Dandong, with lower average annual incidence rates in Jinzhou, Huludao, Fuxin, and Shenyang. We analyzed the possible reasons for this as follows: (1) Topography and terrain: Dalian and Dandong are located in the eastern part of Liaoning Province, mostly in mountainous and hilly areas; Panjin and Shenyang are located in the central part of Liaoning Province, where the terrain is mainly plain, (2) Climate: Dandong is known as the warmest and wettest place in Northeast China because of its unique geographical location. It is also the area with the most rainfall in the north, and Dalian has the highest temperature in the province region. Cities with lower incidence, such as Jinzhou, Huludao, Fuxin, and Shenyang, have lower temperature and drier climate. (3) Percentage of farmer occupations: Dalian and Dandong are in the top two in the province, while Fuxin, Huludao, and Panjin have a lower percentage of farmer occupations. According to the median time of the date of illness onset to the date of diagnosis of SFTS cases, the interval between the onset and diagnosis was > 1 week, which suggested that the primary-level health departments of exceptionally high epidemic areas should enhance health education and improve SFTS surveillance.

Previous studies have shown that the relationship between meteorological factors and SFTS is nonlinear [18]. The GLM extends ordinary linear regression by establishing the relationship between the mathematical expectation of the response variable and the predictor variables through a linkage function. The GLM based on Poisson distribution can directly solve the problem of the nonlinear relationship between meteorological factors and infectious diseases [33]. As the correlation coefficients between the number of lagged SFTS cases and meteorological factors did not change significantly compared to the immediate SFTS cases, we analyzed only the regression relationship between the immediate SFTS cases and meteorological factors. According to the stepwise regression results, monthly average air pressure, monthly average temperature, monthly average wind speed, and time sequence influence SFTS. This process eliminates the co-integrating factors to avoid the problem of multiple co-integration. The distribution of the ICC and the *F*-test showed that the model is highly accurate and the incidence of SFTS were fitted well.

Temperature has always been an important factor affecting the occurrence and development of infectious diseases, and many previous studies have shown that SFTS is closely related to temperature. Sun et al. reported a nonlinear relationship between temperature and SFTS [20], while Zhang et al. found that the maximum temperature in the warmest month is a crucial environmental factor for the incidence of SFTS [19]. We also observed that the peak of the reported cases was consistent with the peak of average temperature in this study. The influence of temperature on the number of cases can be explained from three aspects: First, the temperature can affect the replication and transmission of SFTSV and its survival and development in both the vector and host [34]. Second, the appropriate temperature is conducive to the reproduction, growth, and development of *H. longicornis*, the vector of SFTS [35, 36]. Indeed, studies have shown that a temperature range of 18–35 °C may increase the population density of ticks [37] and contribute to the further reproduction of their offspring. Third, the temperature suitable for tick growth is also ideal for human activities. Under these temperatures, human outdoor activities will increase together with the opportunity for contact with ticks.

Our study also found that SFTS was negatively associated with monthly average air pressure and monthly average wind speed, consistent with a study in Zhejiang Province [38]. Monthly average air pressure can affect the probing period and survival of ticks. Lower air pressure values increase the questing period of ticks, making low air pressure more suitable for ticks to survive [38]; this increases the likelihood of human infestation with ticks and consequently the number of SFTS cases. The negative correlation between monthly average wind speed and SFTS cases may be explained by the fact that wind speed affects tick survival conditions. Higher wind speeds replace forest air (the air in forest that is not affected by other factors) with dry air and accelerate the continued diffusion of carbon dioxide, which acts as an attractant for ticks, resulting in higher tick mortality [39].

## Conclusion

In conclusion, this study comprehensively analyzed the correlation between various meteorological factors and SFTS incidence. We found that monthly average air pressure, monthly average temperature, and monthly average wind speed affected the incidence of SFTS, which provides epidemiological evidence for areas in which tick surveillance is not conducted.

## Supplementary Information

**Additional file 1: Figure S1.** The correlation between the meteorological factors and the number of immediate SFTS cases, Liaoning Province, China, 2011–2019. (**a**) The relationship between monthly average wind speed and the number of immediate SFTS cases. (**b**) The relationship between monthly average relative humidity and the number of immediate SFTS cases. (**c**) The relationship between monthly average sunshine duration and the number of immediate SFTS cases. (**d**) The relationship between monthly average temperature and the number of immediate SFTS cases. (**e**) The relationship between monthly average ground temperature and the number of immediate SFTS cases. (**f**) The relationship between monthly average air pressure and the number of immediate SFTS cases. (**g**) The relationship between monthly average rainfall and the number of immediate SFTS cases

**Additional file 2: Figure S2.** ICC distribution of the optimal selected GLM

## Data Availability

Additional data are available on reasonable request by email to Xinyu Feng (fengxinyu2013@163.com).
